# Phenotypic signatures and genetic determinants of oxacillin tolerance in a laboratory mutant of *Staphylococcus aureus*

**DOI:** 10.1371/journal.pone.0199707

**Published:** 2018-07-03

**Authors:** Marilyn Chung, Vitor Borges, João Paulo Gomes, Herminia de Lencastre, Alexander Tomasz

**Affiliations:** 1 Laboratory of Microbiology & Infectious Diseases, The Rockefeller University, New York, New York, United States of America; 2 Bioinformatics Unit, Department of Infectious Diseases, National Institute of Health, Lisboa, Portugal; 3 Laboratory of Molecular Genetics, Instituto de Tecnologia Química e Biológica António Xavier (ITQB) da Universidade Nova de Lisboa, Oeiras, Portugal; Kent State University, UNITED STATES

## Abstract

Addition of β-lactam antibiotics to growing cultures of bacteria inhibit synthesis of the bacterial cell wall peptidoglycan accompanied by killing (loss of viable titer) and lysis (physical disintegration) of the cells. However, it has also been well established that these antibiotics are not effective in killing non-growing or slow-growing bacteria and the mechanism of this “antibiotic tolerance” is not well understood. In this study, we report on the genetic basis and phenotypic properties of an antibiotic tolerant derivative of the methicillin susceptible *S*. *aureus* strain 27s. Cultures were exposed to “pulses” of high concentrations of oxacillin followed by outgrowth of the surviving bacteria. This procedure quickly selected for antibiotic tolerant mutants with an increased ability to survive antibiotic treatment without increase in the MIC value for the antibiotic. Such mutants also exhibited longer lag phase, decreased lysis, virtually no change in antibiotic susceptibilities, cross tolerance to D-cycloserine and vancomycin, and increase in biofilm formation in the presence of high concentrations of oxacillin. Whole genome sequencing showed that these altered properties were linked to mutations in the *atl* and *gdpP* genes.

## Introduction

*Staphylococcus aureus* is a major pathogen and methicillin resistant strains of *S*. *aureus* (MRSA) are often the primary cause of infections in hospital and clinical settings [1]. Antibiotics such as beta lactams have been developed to treat such infections. In *S*. *aureus* resistance to beta lactams such as methicillin or oxacillin arose through the acquisition of a foreign piece of DNA called the SCC*mec* cassette, which carries the methicillin resistance determinant *mecA* encoding the penicillin binding protein (PBP)2A –a key component of the resistance mechanism in most MRSA isolates [2]. Generally, MRSA infections are dangerous and are the main focus of concern. However, methicillin susceptible *S*. *aureus* (MSSA) are also pathogenic and can acquire tolerance to β-lactams, i.e., the capacity to survive treatment with antibiotics. In antibiotic tolerance, susceptibility (i.e, MIC value) does not change but the bacteria are able to survive the killing action of the antibiotic [3].

In an investigation by Griffith and O’Neill [4], *S*. *aureus* SH1000 was used as the parental strain for isolating a mutant that was tolerant to both oxacillin and vancomycin and was found to have a mutation in the *gdpP* gene [4]. Strain SH1000, a sub-lineage of strain 8325–4 [5, 6], contains a 63 bp deletion in the *spa-sarS* intergenic region that was demonstrated to greatly affect protein A transcription and *sarS* mRNA levels [7]. Protein A is a 42 kDa immunoglobulin binding protein found in the cell wall of *S*. *aureus* and was shown to promote bacterial aggregation and the formation of biofilms [8]. Strains of the 8325–4 lineage are also missing phenol soluble modulin α3 (PSMα3), a virulence determinant linked to neutrophil chemotaxis, biofilm formation and surface spreading [5]. SH1000 is not considered a representative model for in vivo studies because it does not carry protein A unlike most clinical isolates.

In the study described here, a derivative of strain NCTC8325 named strain 27s which contains an intact *spa-sarS* intergenic region was used to obtain an oxacillin tolerant mutant.

## Materials and methods

### Strains and culture conditions

*S*. *aureus* parental strain 27s (also referred to as RN27) was provided by R. Novick, Public Health Research Institute, New York, NY. Strain 27s/RN27 (ST8, *spa* type t211) is lysogenic for phages Φ13 and 80α and was derived from *S*. *aureus*, strain RN25. In turn, strain RN25 was derived by UV treatment from strain NCTC8325 (RN1) [9]. *S*. *aureus* tolerant mutants were obtained from strain 27s by the protocol described below. *S*. *aureus* ATCC25923 and *Staphylococcus epidermidis* strain ATCC12228 were used as controls in some of the assays. *S*. *aureus* strain 27sAtl contains a mutated *atl* gene obtained by transduction with phage 80α from the autolysis-deficient mutant RUSAL2 [10].

All strains were stored at -70°C in tryptic soy broth (TSB; Difco Laboratories, Detroit, Mich.) containing 10% glycerol. Overnight cultures were grown by inoculating 50 μl of the bacterial stocks into 5 ml of tryptic soy broth and incubating at 37°C in glass tubes with aeration overnight. All experiments were done with exponentially growing cultures in mid-log phase obtained by inoculating 0.2 ml of the overnight culture into fresh pre-warmed TSB (50 ml) and cultivation at 37°C under aerobic conditions. Bacterial growth was monitored by measurement of the OD at 620 nm (OD_620_) with a spectrophotometer (Pharmacia LKB Novaspec II, Piscataway, NJ, USA).

### Selection of mutants tolerant to oxacillin

**Fig 1:**
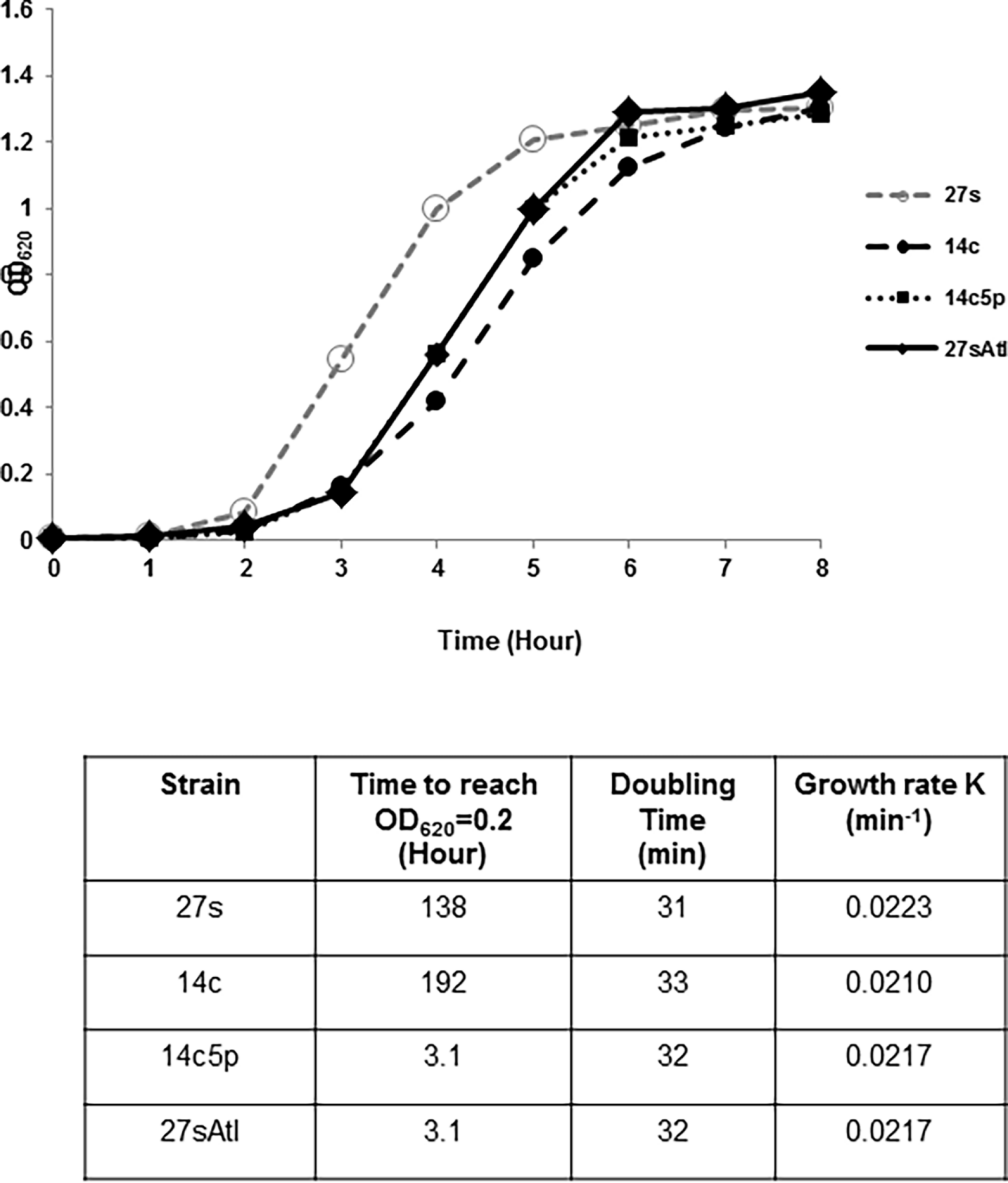
Growth curves of *S*. *aureus* parental strain 27s, tolerant mutants 14c, 14c5p and 27sAtl. Bacterial cultures were grown in TSB at 37°C with aeration.

**Fig 2:**
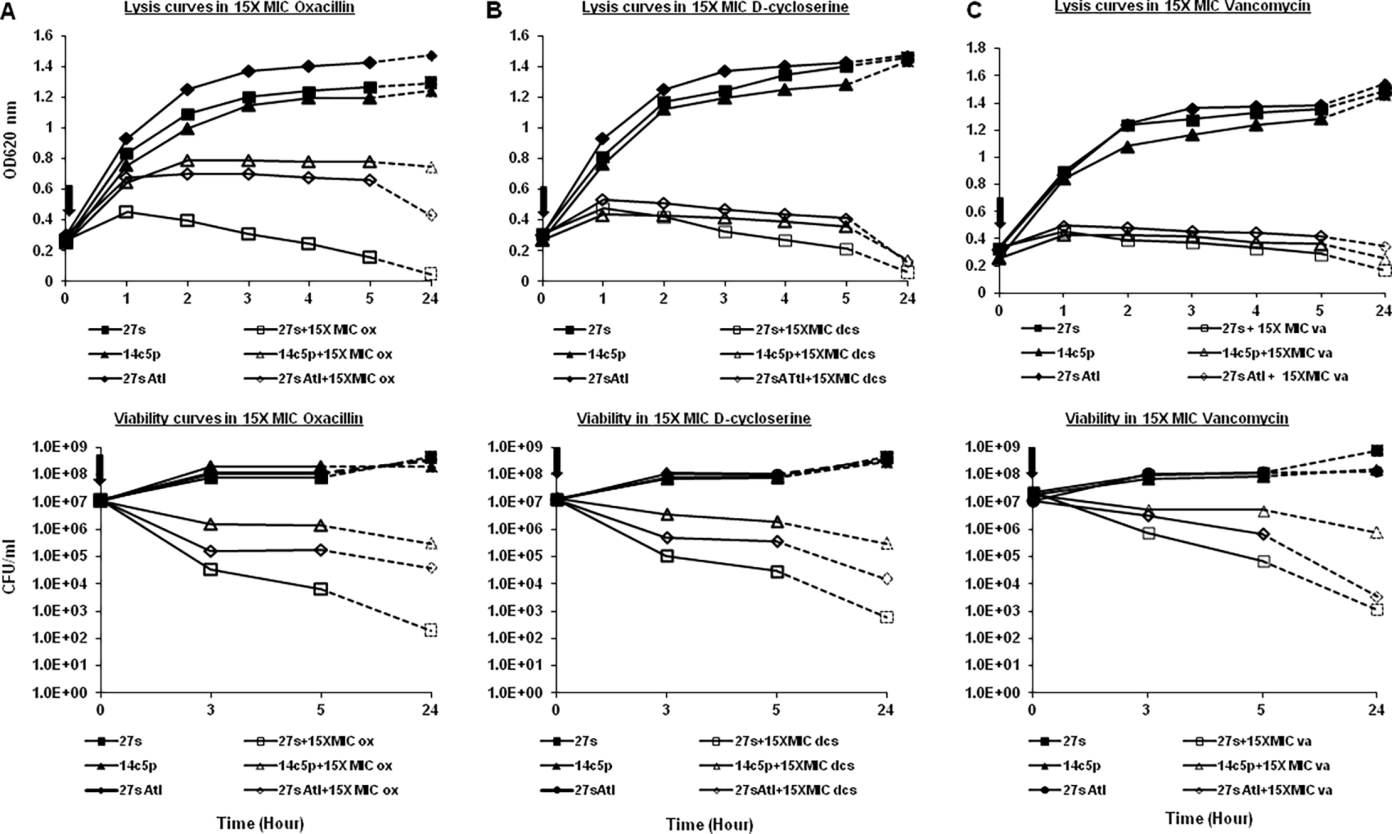
Growth of strain 27s and mutants 14c, 14c5p and 27sAtl in the presence of antibiotics. Antibiotics were added at the time indicated () to the cultures (100 ml) of the exponential phase bacteria (OD620 = 0.2–0.4 nm). The antibiotics tested and the concentrations used were: (A) oxacillin (15 X MIC, 1.5 mg/L), (B) D-cycloserine (15 X MIC, 22 mg/L) and (C) vancomycin (15 X MIC, 15 mg/L). The top panel follows the lysis of the cultures by reading the OD620 after the addition of the antibiotic at time 0 and every hour for 5 hours and then after 24 hours. The bottom panel shows the effect of antibiotics (A) oxacillin, (B) D-cycloserine and (C) vancomycin at the concentration of 15 X MIC on the viability of the wild type 27s and mutant strains. Viability was determined by serial dilutions of aliquots taken at 0, 3, 5 and 24 hours onto tryptic soy agar plates and counting the CFU’s after 24 hour incubation of the plates at 37°C.

A mutant tolerant to oxacillin was obtained by cyclic exposure of strain 27s to “pulses” of high concentration of oxacillin (15X MIC). First, 50 μl of an overnight culture of 27s grown in TSB was inoculated into 50 ml of fresh pre-warmed TSB. Oxacillin at 15 X MIC (1.5 mg/L) was added to the culture when the OD620 nm reached 0.2–0.4. Measurements of the OD620 were taken every hour and aliquots of 1 ml were removed at 0, 3, 5 and 24 hours to assay bacterial survival (Figs 1 and 2A–2C).

From the aliquot taken at 5 hours, 100 μl of the washed, antibiotic free suspension was inoculated into 5 ml of TSB and incubated overnight with aeration at 37°C. The overnight culture was diluted the next morning in fresh TSB and oxacillin at 15 X MIC (1.5 mg/L) was added when the culture reached mid-log phase. Every time this process was repeated it will be referred to as a “cycle”. A mutant “tolerant” to oxacillin was obtained after 14 cycles of treatment with the antibiotic (mutant 14c).

### Growth curves

Overnight cultures of strain 27s and the tolerant mutant were diluted into fresh prewarmed TSB and absorbance at OD620 nm was measured every 60 minutes. The measurements were plotted and the doubling times were calculated from the graph. Growth rates were calculated by the formula *k = log(X*_*t*_*)–log(X*_*0*_*) / 0*.*301t* (see Fig 1).

### Antibiotic-induced lysis

To assess antibiotic-induced lysis, antibiotics (oxacillin, vancomycin or D-cycloserine) were added at concentrations of 15 times the MIC when the culture (at 37°C) reached an absorbance of about 0.2 to 0.4 (corresponding to 10^7^ to 10^8^ log10 CFU/ml). The absorbance at OD620 nm was measured every 60 minutes for five hours after the addition of antibiotic and also after 24 hours (Fig 2A–2C).

### Viability of strains after addition of antibiotic

To determine the number of survivors of a strain after treatment with 15X MIC of the antibiotic, aliquots of 1 ml were removed at 0, 3, 5 and 24 hours. The 1 ml aliquots were centrifuged in eppendorf tubes for 5 minutes at 13,000 rpm. The supernatant was discarded and the pellet was suspended with 1 ml of TSB. The pellet was washed 3X with 1ml of TSB to remove the antibiotic, suspended with 1 ml of TSB and diluted to -1, -2,-3,-4, -5 and -6 with TSB. 100 μl from each dilution were plated on TSA plates and incubated at 37°C overnight. The CFU were counted and the number of survivors determined for each strain as the log CFU reduction between the control and the 3, 5, and 24 h culture in the presence of antibiotic (Fig 2). Log reductions were calculated according to http://microchemlab.com/log_reduction_and_percent_reduction_calculations.

### Testing for stability of 14^th^ cycled oxacillin mutant (mutant 14c)

The 14^th^ cycled oxacillin tolerant mutant was streaked on a TSA plate and incubated at 37°C overnight. A single colony was inoculated into 5 ml antibiotic free TSB and incubated at 37°C with rotation for 18 hours. After 18 hours, 10 μl of the overnight culture was diluted into fresh pre warmed TSB. This process was repeated 4 more times for a total of five passages of the 14^th^ cycled oxacillin mutant (14c5p)

### Antibiotic susceptibility

All strains were tested for antimicrobial susceptibility by disk diffusion, Etest and Population Analysis Profiles.

The standard disc diffusion test on Tryptic Soy Agar was performed as follows: overnight cultures were inoculated into TSB and incubated at 37°C until they reached an OD620 equal to a MacFarland 0.5 standard. Bacterial cultures were then plated onto TSA and antibiotic disks (BD, BBL, Franklin Lakes, NJ) were placed on the plates. The plates were incubated at 37°C for 18–24 hours. The diameters (in millimeters) of clear zones were measured. The antibiotic sensi disc (BD, BBL, Sensi-Disc, Becton Dickinson and Company, USA), with the concentrations that were tested included: penicillin G (10 μg), ciprofloxacin (5 μg), erythromycin (15 μg), gentamicin (10 μg), linezolid (30 μg), nitrofurantoin (300 μg), rifampin (5 μg), and tetracycline (30 μg). Each strain was classified either as Resistant(R), Intermediate (I) or Susceptible (S)–according to guidelines developed by the Clinical and Laboratory Standards Institute recommendations [11].

**Fig 3:**
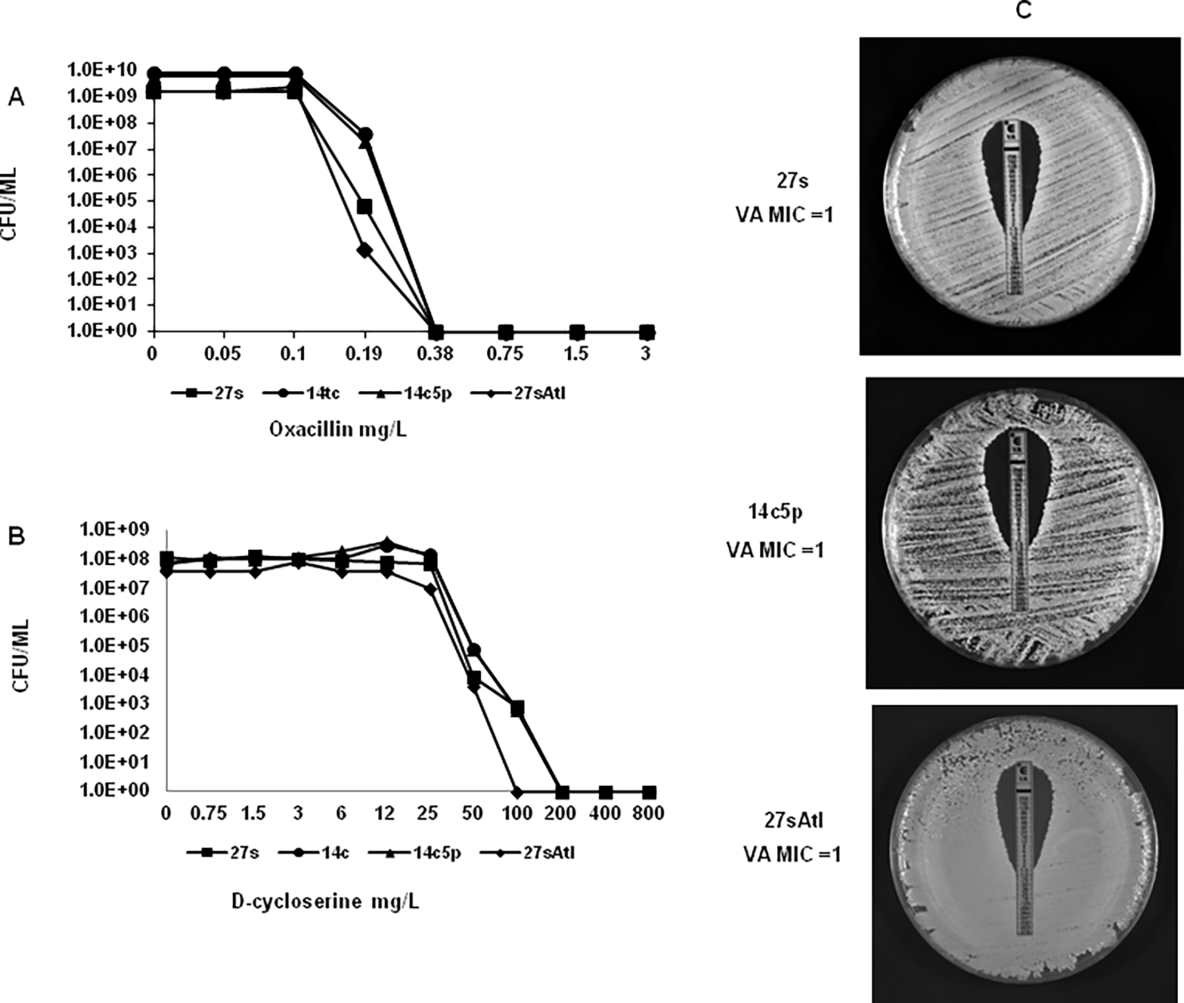
Population analysis profiles and Etest of parental strain 27s, and mutants. Stationary phase cultures of 27s, 14c, 14c5p and 27sAtl were plated with several dilutions on agar plates containing increasing concentrations of (A) oxacillin or (B) d-cycloserine (C) Vancomycin susceptibility of 27s, 14c5p and 27sAtl were determined by Etest.

The Etest was performed by spreading a small aliquot of overnight culture diluted to an OD620 of 0.08 onto TSA plates and adding an Etest strip of oxacillin, vancomycin, daptomycin, cefoxitin or daptomycin onto the surface of the plate. MIC values of the antibiotic were determined after 24 hour incubation at 37°C (Fig 3).

PAPs were done on overnight cultures diluted with TSB and plated on TSA plates containing serial (2 fold) dilutions of oxacillin, vancomycin or D-cycloserine according to the population analysis method as previously described [12] (Fig 3).

### Biofilm formation

**Fig 4:**
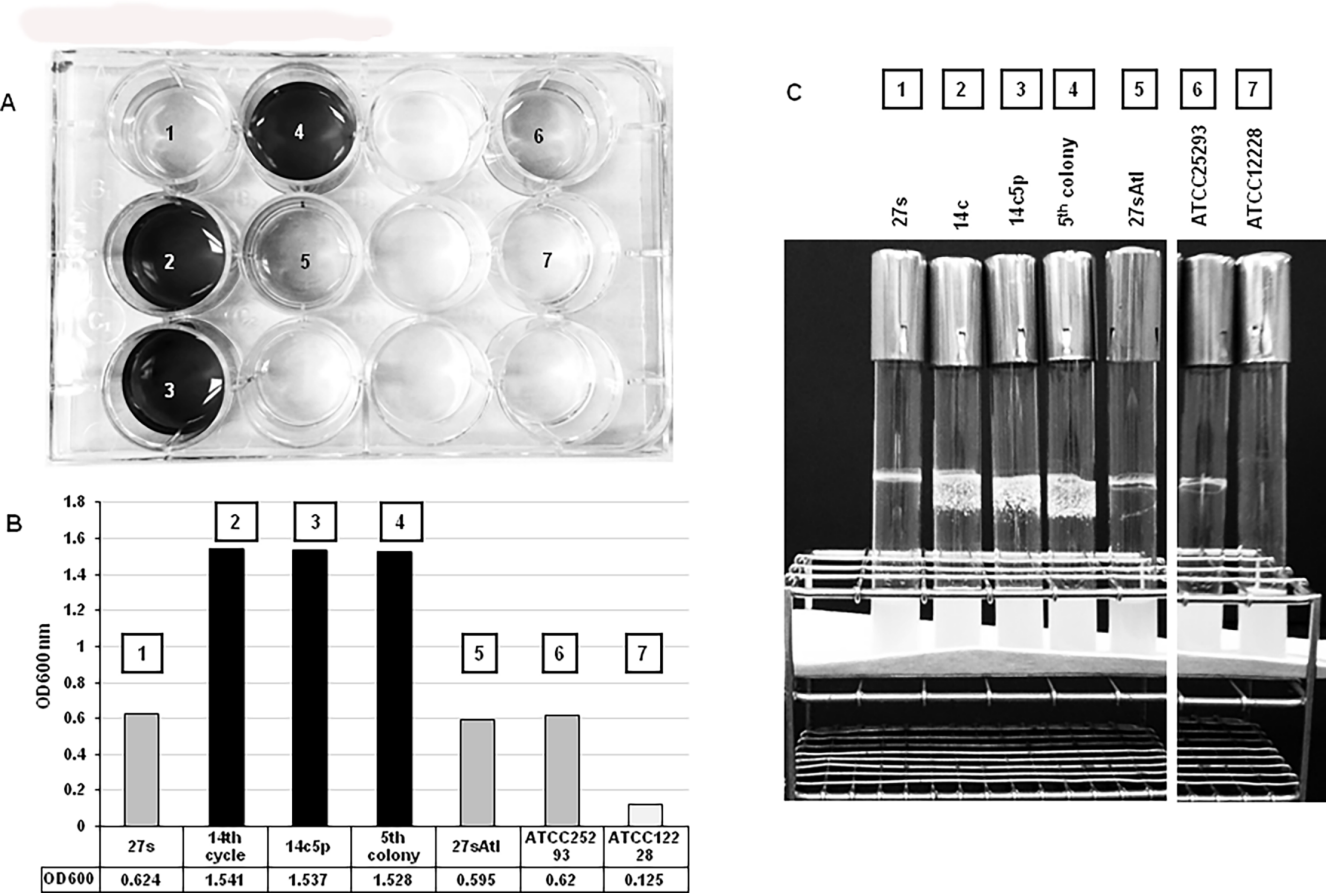
Comparison of biofilm formation between 27s, tolerant mutants and control strains. **(A)** in plastic 96-well plates measured by the crystal violet assay; **(B)** Spectrophotometric values at OD600nm quantifying the recovery of crystal violet from stained biofilms; **(C)** Biofilm formation in TSB medium with 20^0^/_0_ glucose after 24 hours incubation at 37°C with rotation.

The biofilm assay was done by an adaptation of a protocol using crystal violet [13, 14]. Parental wild type *S*. *aureus* strain 27s, tolerant mutants 14c and 14c5p and control strains ATCC25923 and ATCC12228 were inoculated into 5 ml of TSB and incubated at 37°C with aeration overnight. The overnight cultures (50 μl) were diluted into 50 ml of pre-warmed TSB with 2% (w/v) glucose to maximize *ica* operon induction, as reported [15] and incubated at 37°C with agitation until the culture reached an OD_620_ = 0.5–0.7. Aliquots (4 ml) of each culture were added to 6 well plates and incubated at 37°C for 24 hours (Fig 4A). After incubation, 3.8 ml of the culture fluids from each well were carefully aspirated, the plate inverted and blotted on paper towels 3X. In order to remove the unattached cells and media components, 4 ml of sterile distilled water was added into each well. The water wash was aspirated and the plate blotted on paper towels 3X and was repeated twice. Crystal violet solution (4 ml of 0.1%) was added to each well and the plates were incubated at room temperature for 15 minutes. The crystal violet solution was removed by aspiration and the wells were rinsed 3 X with 4 ml sterile distilled water. The plate was blotted 3 X on paper towels. The plate was then incubated at 80°C for 1 hour to fix the stain. Next 4 ml of 10% SDS in 40% ethanol was added to each well. The plate was left at room temperature for 30 minutes. 1 ml of the solubilized crystal violet from each well was transferred to a Sarstedt cuvette and the absorbance at OD_600_ nm was measured using a spectrophotometer (Fig 4B).

### Biofilm formation in glass test tubes

Strains 27s, the tolerant mutants 14c and 14c5p, strain 27sAtl and controls ATCC25923 and ATCC12228 were streaked on TSA plates. The plates were incubated at 37°C overnight for 18 hours. A single colony from each strain was inoculated into glass test tubes containing 6 ml TSB with 2% (w/v) glucose and incubated with rotation at 37°C for 18 hours (Fig 4C).

### Whole-genome sequencing

Genomic DNA was extracted from the parental strain 27s and from the oxacillin tolerant mutant 14c5p using the Qiagen DNeasy Blood & Tissue Kit (QIAGEN, Ambion Inc., Austin, TX, USA). Paired-ended (2 x 250 bp) sequencing was performed at the Instituto Gulbenkian de Ciência (IGC, Oeiras, Portugal) using the Illumina MiSeq platform. Quality control of reads, *de novo* assembly and contigs quality assessment, and possible contamination search was carried out using the multi-software pipeline INNUca version 2.6 (https://github.com/B-UMMI/INNUca). Selected assemblies for parental isolates were inspected and subsequently annotated using Prokka (https://github.com/tseemann/prokka) (version 1.12). Mean depth of coverage (post quality control) ranged from 98- and 88-fold for 27s and 14c5p, respectively. Quality processed reads of 14c5p were mapped against the corrected assembly of the parental strain using Snippy v3.1 (https://github.com/tseemann/snippy) with the following criteria: i) minimum mapping quality of ≥20; ii) minimum number of 10 quality processed reads covering the variant position; iii) a minimum proportion of 90% of quality processed reads at the variant position differing from the reference. All mutations were inspected and confirmed using Integrative Genomics Viewer (http://software.broadinstitute.org/software/igv/).

### Read sequences for the isolates used in this study

The sequences for the *S*. *aureus* isolates are in the Sequence Read Archive (SRA) under the Bioproject (http://www.ebi.ac.uk/ena/data/view/ERS2296040 and http://www.ebi.ac.uk/ena/data/view/ERS2296041) and can be downloaded from the NCBI’s website (http://www.ncbi.nlm.nih.gov/).

## Results

In a search for the genetic basis and phenotypes of βlactam tolerance, we isolated a tolerant mutant, designated as 14c5p [i.e., obtained after five passages of a 14^th^ cycled oxacillin mutant (14c)], from the parental strain 27s, which is a derivative of the MSSA strain NCTC8325. Comparison at the whole-genome level revealed only two mutations in the tolerant strain: one inactivating mutation in the *atl* gene, which encodes a bifunctional autolysin [16] and a missense mutation in the *gdpP* gene, which encodes the cyclic-di-AMP phosphodiesterase GdpP (Table 1). Whereas GdpP is already known to mediate tolerance to βlactams and glycopeptides [4], no direct link has been established as yet for *atl*. We thus generated an *atl* truncated mutant (27sAtl) to investigate potential changes in phenotypic properties.

**Table 1:** Mutations found in the tolerant mutant 14c5p.

Mutation	Effect	Nucleotide	Amino Acid	Gene	Locus tag	Product
number		change	change		in reference*	
1	stop_gained	2194C>T	Gln732*	*atl*	SAUSA300_0955	Bifunctional autolysin
2	missense_variant	1301C>A	Ala434Glu	*gdpP*	SAUSA300_0014	Cyclic-d-AMP phosphodiesterase GdpP

*According to the genome annotation of the reference strain USA300_FPR3757 (accession number CP000255) [17]

### No change in antibiotic susceptibilities

The tolerant mutants 14c and 14c5p remained susceptible to all eight antibiotics tested by disk diffusion and Etest (results not shown). The population analysis profiles (PAPs) of the tolerant mutant for oxacillin, vancomycin and D-cycloserine were virtually identical to that of the parental strain 27s (Fig 3).

### Prolonged lag phase before exponential phase

A unique property of the tolerant laboratory isolate was the prolonged lag (186 min) before resumption of exponential growth when stationary phase overnight cultures were diluted into fresh medium (Fig 1). The doubling time of tolerant mutants 14c and 14c5p was 32 minutes about the same as that of 27s (31 minutes) in the exponential growth phase. The 27s Atl mutant showed a similar lag time of 186 minutes and doubling time of 32 minutes (Fig 1).

Oxacillin induced lysis of the tolerant mutant 14c5p was much slower than that of the parental strain 27s (Fig 2A). When 15 x MIC (1.5 mg/L) oxacillin was added to mid log cultures of the parental strain 27s and the tolerant mutant at the OD620 = 0.3, the 27s culture grew only to an OD620 of 0.539, while the tolerant mutant was able to grow up to OD620 = 0.798. The optical density OD620 nm of the 27s culture started to decrease at OD620 = 0.53, i.e., 3 hours after the addition of oxacillin to the medium, while the tolerant mutant showed no decrease in optical density (at OD620 = 0.798) during the same time period. After overnight incubation of the cultures with oxacillin, the parental strain 27s showed almost complete lysis while the optical density of the tolerant mutant did not decline.

There was only a 2 log reduction in survivors for the tolerant mutant culture after 3 hours of exposure to 15X MIC oxacillin as compared to a 6 log reduction in the number of survivors of the parental culture (Fig 2A). After 24 hours exposure to high concentrations of oxacillin, the tolerant mutant had a 5 log reduction in viability, while the parental culture had a 7 log reduction in viability.

The phenotypes of longer lag phase, decreased lysis and increase in the number of survivors in the exponential phase was stable in the oxacillin tolerant mutant after five passages in antibiotic free TSB (14c5p) (Fig 2).

### Increase in biofilm production

*S*. *aureus* exposed to low concentrations of beta lactam antibiotics such as oxacillin can induce the formation of biofilm [17]. Biofilm assays showed that the 14c mutant and its 14c5p derivative produced 2.5 times the amount of biofilm as compared to the 27s parental strain. A huge ring of biofilm was observed inside the surface of the glass test tube when 14c and 14c5p were grown in TSB (Fig 4C). Aggregate formation was also observed at the bottom of the flasks for 14c and 14c5p, but not in strains 27s and 27sAtl (not shown).

### Cross tolerance to other antibiotics

The 14^th^ cycled mutant displayed cross-tolerance to the glycopeptide antibiotic vancomycin. The vancomycin MICs of strains 27s and the oxacillin tolerant mutant 14c5p were both 1 mg/L by Etest. The oxacillin tolerant mutant exhibited a reduction in killing compared with 27s after 3 and 5 hours exposure to 15 X MIC (15 mg/L) of vancomycin. There was a two-log reduction in survivors in the 27s culture as compared to one log reduction for the 14c5p mutant after 3 hours exposure, while there was a three log reduction for strain 27s and a one log reduction for the 14c5p mutant after 5 hours (see Fig 2).

Mutant 14c5p also showed tolerance to D-cycloserine. The D-cycloserine MIC of strain 27s and the mutant were 25 mg/L each. There were more survivors in the mutant during the exponential growth period than in 27s when treated with 15X MIC of D-cycloserine (22 gm/L). There was a three log reduction for the 27s and a one log reduction for the mutant 14c5p after 3 and 5 hours treatment with the antibiotic (see Fig 2).

The 27sAtl mutant also showed tolerance to both oxacillin and D-cycloserine, and slight tolerance to vancomycin. The oxacillin MICs of strains 27sAtl and 27s were both 0.125 mg/L by Etest. The D-cyloserine MIC for 27sAtl and 27s were both 25 mg/L by PAP. The lag period of 27sAtl mutant (186 minutes) was longer than the lag phase of the 27s strain (138 minutes) (Fig 1). There was a three-log reduction in the number of survivors after 3 hours treatment of 27sAtl with 15X MIC oxacillin and a six log reduction in the number of survivors for 27s. There was a four-log reduction for 27sAtl and a six-log reduction for 27s after 5 hours exposure to 15 X MIC oxacillin. There was a two-log reduction in the number of survivors when 27sAtl was treated with 15X MIC D-cycloserine (375 mg/L) compared to three-log reduction for 27s after 3 hours. After 5 hours, there was still a two-log reduction for 27sAtl and a three-log reduction for 27s treated with 15X MIC D-cycloserine. There was a slight tolerance to vancomycin when 27sAtl was treated with 15X MIC vancomycin (22.5 mg/L) as compared to 27s and less tolerance than in mutant 14c5p (see Fig 2).

## Discussion

Whole genome sequencing of the oxacillin tolerant mutant described in this study identified mutations in two genes when compared to the non-tolerant parental strain: one loss-of-function mutation in the *atl* gene and a non-synonymous mutation in *gdpP*. An earlier study by Griffith et al used SH1000 as the parental strain and identified only a loss-of-function mutation in the *gdpP* gene [4]. This reinforces the role of the latter in antibiotic tolerance and points to *atl* as a putative novel mediator of this phenotype.

Atl, is a 137.5 kDa bifunctional protein with two domains: AM (amidase) and GL (glucosaminidase) that are processed outside the cell and adhere to the staphylococcal surface at specific locations of the equatorial surface rings. [16, 18]. Analysis of the lysis products of *S*. *aureus* peptidoglycan indicates that the enzyme may be classified as an *N*-acetylmuramyl-l-alanine amidase [19]. Atl is involved in the separation of daughter cells during cell division and it also contributes to cell wall turnover and antibiotic-induced lysis [20], which are mechanisms that may support the phenotypes observed in our study, namely that the inactivation of the *atl* gene in parental strains has led to extended lag phase and cross tolerance to several antibiotics.

One virulence property of *S*. *aureus* is the capacity to form biofilm which involves two processes: attachment to a surface and cell clustering abetted by a slimy material called the polysaccharide intercellular adhesin, PIA [21]. Atl deletion mutants were shown to have decreased biofilm formation [19]. Intriguingly, in our study, the 14c5p mutant produced 2.5 x more biofilm than the parental strain 27s, and yet the 27sAtl mutant had the same amount of biofilm as the parental strain–as determined by the crystal violet stain assay. The lack of impact of the deletion of *atl* on the biofilm phenotype has been shown previously [22]. Mutant 27sAtl did not form aggregates nor showed the stickiness to the surfaces of glass tubes–in contrast to strain 14c5p grown in TSB. As such, the biofilm-associated phenotype observed for 14c5p is likely provided by the non-synonymous mutation in *gdpP*.

GdpP (GGDEF domain protein containing phosphodiesterase) is a phosdiesterase that degrades c-di-GMP and c-di-AMP in bacteria such as *S*. *aureus*. Cyclic diadenosine monophosphate (c-di-AMP) is synthesized from two molecules of ATP by the diadenylate cyclase enzyme, DacA and is degraded into pApA by GdpP [23, 24]. C-di-AMP is a signaling molecule and is a second messenger in *S*. *aureus*. It has been shown that deletion of *gdpP* in *S*. *aureus* increases intracellular levels of c-di-AMP and decreases the size of the bacterial cell. C-di-AMP, stimulates the biosynthesis of adhesins and promotes biofilm formation. Strains with deleted GdpP produce approximately 3X the amount of biofilm as compared to the WT strain [24]. In our study, the 14^th^ cycled oxacillin mutant produced 2.5 X the amount of biofilm as compared to the parental strain 27s. Considering that this mutant carries a non-synonymous mutation in *gdpP* (while mutants in other studies had *gdpP* deleted), one may speculate that non-inactivating mutations may be sufficient to reduce the phosdiesterase activity, yielding a cascade of phenotypes: accumulation of C-di-AMP, promotion of biofilm formation and, ultimately, antibiotic tolerance. Indeed, an increase of biofilm formation can slow the penetration of β-lactams and aminoglycosides in bacteria giving the bacteria a longer survival period [25]

The oxacillin tolerant mutants 14c and 14c5p described in this communication and strain 27sAtl each showed cross tolerance to D-cyloserine and vancomycin. In the study by Griffith et al, deletion of GdpP in SH1000 oxacillin tolerant mutant also showed cross tolerance to vancomycin [4]. D-cycloserine is a cyclic analog of D-alanine that blocks the early cytoplasmic phase of peptidoglycan synthesis by inhibiting D-alanyl-D-alanyl ligase (Ddl). On the other hand, vancomycin is a glycopeptide antibiotic which inhibits cell wall synthesis by binding the D-ala-D-ala of the lipid II and sterically hindering transglycosylation and transpeptidation. Similarly to oxacillin, vancomycin blocks the late stage of peptidoglycan biosynthesis by inhibiting the action of PBPs that cross-link the immature peptidoglycan [26]. In this context, our observation that cross tolerance can involve antibiotics targeting both the early (D-cycloserine) and late stages (vancomycin and oxacillin) of peptidoglycan synthesis further supports that the *gdpP/atl*-mediated mechanisms of tolerance are beyond the specific molecular pathways where antibiotics are known to act. Considering that 27sAtl mutant displayed both vancomycin and D-cycloserine tolerance, but these phenotypes were less marked than the ones observed for the 14c5p strain (double mutant), it is reasonable to hypothesize that the enhanced phenotype in 14c5p relies on the synergistic effect of *atl* and *gdpP* mutations.

In summary, an oxacillin tolerant *S*. *aureus* laboratory mutant described here has several unique properties including longer lag phase; decreased lysis; increase in the amount of survivors in the exponential phase; virtually no change in antibiotic susceptibilities, cross tolerance to D-cycloserine and vancomycin and increase in biofilm formation in the presence of high concentrations of oxacillin. These phenotypes can be linked to *gdpP* gene, through either its deletion (previous studies) or discrete mutations (our study), and to the inactivation of *atl* gene, a potential novel mediator of *S*. *aureus* ability to acquire antibiotic tolerance during the course of treatment.
